# Theory of Mind Development in Deaf and Hard-of-Hearing Individuals: A Systematic Review

**DOI:** 10.3390/bs15081065

**Published:** 2025-08-06

**Authors:** Leire Martín, Mario Figueroa, Beatriz de Diego-Lázaro, Raquel Balboa-Castells, Gary Morgan

**Affiliations:** 1 Department of Psychology and Education, Universitat Oberta de Catalunya (UOC), 08018 Barcelona, Spain; rbalboa@uoc.edu (R.B.-C.);; 2 Department of Basic, Developmental and Educational Psychology, Universitat Autònoma de Barcelona (UAB), 08193 Barcelona, Spain; mario.figueroa@uab.cat; 3 Department of Cognition, Development, and Educational Psychology, Universitat de Barcelona (UB), 08035 Barcelona, Spain; 4 Institute of Neurosciences, University of Barcelona, 08035 Barcelona, Spain; 5 Department of Educational Psychology, University of Valladolid, 40400 Valladolid, Spain

**Keywords:** deaf, hard of hearing, Theory of Mind, false belief, signed language, cochlear implant, parent–child interactions

## Abstract

Theory of Mind (ToM) is a construct that includes a range of connected abilities linked to the understanding of others’ mental states. During the last three decades, ToM development has been studied extensively in deaf and hard of hearing (DHH) individuals and performances compared to the typically hearing (TH) population. Given the advances in the early diagnosis of deafness, interventions, and hearing devices over this period, variations in task performance among DHH participants might have been reduced. The current systematic review aims to synthesize all studies of ToM in DHH individuals and answer the following question: Do DHH individuals (Population), compared to a control sample of TH and/or among themselves (Comparator), in an assessment of ToM (Intervention), have differentiated results (Outcome)? After a search of the literature, 97 papers were included. We found that, in general, TH participants outperformed their DHH peers in ToM measures; however, there was a wide range of results. Explanations for this variability included the quality of early interactions and early exposure to both signed and spoken language. The review also indicates that the understanding of false belief was the most studied component within ToM, while other components, such as understanding intention and irony, require further research. Implications of these findings for clinical practice are discussed.

## 1. Introduction

### 1.1. Theory of Mind

Theory of Mind (ToM) allows people to infer and understand mental states, as well as, attribute desires, emotions, and beliefs to themselves and others in order to anticipate intentions, behaviours, and emotions (Premack & Woodruff, 1978; Shahaeian et al., 2011; Seidenfeld et al., 2014). Westby and Robinson (2014) write that ToM involves cognitive, affective, and social factors. ToM is also a developmental construct comprising interconnected levels that emerge from early childhood to young adulthood (Fu et al., 2024; Peterson et al., 2012; Pons et al., 2004; Wellman & Liu, 2004; Westby & Robinson, 2014). The most well-known model of this progression is the six-step ToM scale (Peterson et al., 2012), which assesses the understanding of increasingly complex components: diverse desires, diverse beliefs, knowledge access, false belief, hidden emotions, and sarcasm. Research has showed that any delays in ToM development have a negative impact on children’s academic achievement (Aydın & Özgeldi, 2020; Bernier et al., 2023), emotional processes, and social relationships (Baker & Crnic, 2009; Caputi et al., 2012).

### 1.2. ToM Development in Typically Hearing Individuals

Research on the development of ToM proposes ages at which most typically developing children acquire the different components. Children as young as 18 months understand that humans have goals and intentions in contrast to inanimate agents (Meltzoff, 1995). Intention understanding is the ability to recognize that others act with purpose or goal-directed behaviour (Wellman & Liu, 2004). Toddlers aged 18–24 months and young preschoolers (aged from 2 to 3 years) acknowledge that others may have different desires and emotions and have thoughts and actions based on those (e.g., Gopnik & Slaughter, 1991; Meltzoff, 1995; J. O’Reilly & Peterson, 2014; Poulin-Dubois & Yott, 2018; Wellman & Woolley, 1990; see also the meta-analysis by Wellman & Liu, 2004). These early ToM components (intentions, desires, and emotions) have been assessed through the observation of imitation behaviours (Meltzoff, 1995), eye gaze tracking (Vaish et al., 2018), looking time patterns (Poulin-Dubois & Yott, 2018), and specific tasks from the 6-step ToM scale (Peterson et al., 2012).

One ToM component, false belief (FB), is widely regarded as a crucial milestone in cognitive development. This involves the understanding that others may act based on a misconception. At 1–2 years, young children behave as if they are aware implicitly of FBs (Poulin-Dubois & Yott, 2018); however, it is during the preschool years, from 4 to 6 years old, that children begin to be able to explain explicitly that others can have FBs (e.g., Callaghan et al., 2005; Wellman et al., 2001), and this allows them to attribute beliefs to others that are different from their own. The Sally–Ann task (Baron-Cohen et al., 1985) is the most commonly used standard tool to assess FB and involves an unexpected location change (an object has been moved from its original location without the knowledge of one of the two characters). Individuals are presented with a two-doll story representing a conflict between reality and a character’s mistaken belief. Most 3-year-olds consistently fail these type of FB tests because they do not realize that people do not behave based on reality itself, but on what they believe to be true about reality (Tomasello, 2018) (e.g., the girl will look in the location where she believes an object to be, rather than its true location; Perner & Wimmer, 1985). Another often-used standard FB task involves unexpected contents (e.g., a Smarties tube containing not sweets but a pencil: Perner et al., 1987).

The particular demands that the FB task entails have been described in the ToM literature. In order to follow the standard FB tasks, children must deal with some linguistic and executive function demands. For example, inhibitory control and memory for task-relevant information (Wellman et al., 2001). However, Wellman et al. (2001) noted in their meta-analysis that many researchers have modified standard FB tasks to reduce these executive function demands and ensure that language skills do not impact performance. For example, using the Sally–Ann task, the story can be administered in a minimally verbal way by acting it out with dolls and asking participants to answer the FB questions by pointing to objects. Moreover, one way to reduce memory and inhibitory demands is to remove real-world elements from the story, such as the actual presence of an object. This eliminates the need to suppress knowledge of reality when inferring FBs (e.g., removing the ball from the Sally–Ann story after it has changed location: Wellman et al., 2001). While these task modifications can be helpful to children’s performance, they do not fundamentally change the overall developmental trajectory. With these adaptations, children still do not pass FB tasks until 4–6 years of age.

Older children grasp a more advanced stage of FB, termed second-order FB. This understanding refers to situations where someone may hold a FB about another person’s belief (e.g., John did not know that when he changed the location of the toy, his friend was watching this change through a window; Perner & Wimmer, 1985). Despite passing first-order FB tasks, children often fail second-order FB as late as ages 7 to 9 years. However, there is some disagreement over whether this is related to children’s ToM or still immature executive functioning (Hao & Su, 2014; Liu et al., 2018; Macaulay & Ford, 2013) or not having more advanced language skills, such as the use of recursive complement sentences (J. G. de Villiers et al., 2014; Hollebrandse et al., 2014). Finally, ToM steps beyond beliefs (e.g., understanding sarcasm, lies or morality) require children and adolescents up to 11–12 years to understand that literal meanings of people’s words might not match their intended meanings (Peterson et al., 2016a). The Strange Stories (Happé, 1994) or the *Faux pas* tasks (Baron-Cohen et al., 1999) are used for assessing these advanced ToM components. These stories contain situations from everyday life in which an individual may say something that is not literally true in the case of Strange Stories or is not socially correct in the case of Faux pas. In their systematic review of ToM in typical hearing (TH) children, Beaudoin et al. (2020) found that the different ToM components have received differing levels of attention in the literature, with beliefs and especially FB being over-represented. In 830 studies, 75.5% included a task from the category beliefs, and only 4.3% of papers measured intentions.

### 1.3. Explanations of ToM Development

There is much debate about the factors underlying ToM development and its observed variability across TH children. One theory posits that ToM emerges from early caregivers–child communicative interactions (Tomasello, 2018; Yu & Wellman, 2023) where there is a sharing of an intention, as well as, joint attention on objects (Westby & Robinson, 2014). Connected to this model, Ensor and Hughes (2008) showed that mothers’ engagement in turn-taking with infants at 2 years of age, predicted children’s ToM understanding at the age of 4 years. Furthermore, caregivers’ everyday mental-state talk (Devine & Hughes, 2019; Ruffman et al., 2002) and wider language which includes syntactic complementation (J. G. de Villiers & de Villiers, 2014; Milligan et al., 2007) and diverse vocabulary (Farrar & Maag, 2002) has been connected to ToM development and variability. The advanced components of ToM (such as second-order FB, sarcasm, lies, and morality) have also been linked to older children’s verbal reasoning skills (J. G. de Villiers et al., 2014) and peer social competence (Bosacki & Astington, 1999; Peterson et al., 2016b). Thus, communication and language are seen as very related to ToM skills.

### 1.4. ToM Development in DHH Individuals

Over the past three decades, ToM has been studied across various populations with atypical development, including individuals with autism (Baron-Cohen et al., 1985) and those with mild cognitive disabilities (Benson et al., 1993). Early research also identified congenital deafness as a condition that can significantly affect ToM development (Peterson & Siegal, 1995). Delays in ToM development have been consistently observed among DHH individuals, particularly in the 90–95% (Mitchell & Karchmer, 2004) born into hearing families who often have limited prior knowledge of deafness or signed language at the time of their child’s birth, compared with DHH children with DHH caregivers (Courtin, 2000; Hall et al., 2018; Peterson & Siegal, 1995; Steeds et al., 1997).

Different explanations have been proposed to account for ToM difficulties in DHH individuals, which are rooted in broader developmental models of ToM (Tomasello, 2018; Yu & Wellman, 2023). One prominent line of research attributes ToM delays to limitations in exposure to early quality communicative interactions and language. With regard to communication, hearing caregivers often rely on auditory strategies to gain their child’s attention (Wille et al., 2019) and face challenges in maintaining engagement long enough to support successful interaction (Wille et al., 2019; Kelly et al., 2020; Lieberman et al., 2014), thereby limiting opportunities to provide rich, symbol-based linguistic input (Spencer & Lederberg, 1997). Studies have shown that hearing caregivers of young DHH individuals tend to exhibit less flexibility, sensitivity, positive affect, and responsiveness during interactions, while engaging in more directive communication styles (Lederberg et al., 2013; Spencer & Lederberg, 1997; Wille et al., 2019).

Turning now to delayed language exposure as a potential explanation for ToM challenges, it is noteworthy that hearing caregivers typically use spoken language to convey abstract concepts, such as beliefs and emotions, which can be difficult for DHH children to perceive auditorily during the first years of life. This challenge is further heightened by variability in early access to speech sounds via cochlear implants and hearing aids, which contributes to differences in language exposure (Faistauer et al., 2022; Gordon et al., 2022). There are substantial differences across populations and time-periods in the research literature regarding when hearing devices were introduced and became functional for DHH individuals. A delay in exposure to speech can hinder the development of joint attention, a foundational skill in the progression of ToM (Westby & Robinson, 2014). Krysztofiak and Pluta (2021) conducted a literature review on ToM development in DHH children with cochlear implants, concluding that while these hearing devices can support ToM development, variability in outcomes highlights the importance of early language exposure and continued support with developing interaction. Timely language interventions often lead to improvements in auditory perception, language comprehension, and expressive skills in DHH children, with outcomes approaching those of TH peers (Dettman et al., 2007; McConkey Robbins et al., 2004; Sanju et al., 2022; Yoshinaga-Itano et al., 2017); however, access to spoken language in the first years of life remains inconsistent. On the other hand, exposing children to sign language provides another accessible path to language development. Some hearing families of DHH children successfully learn sign to support their child’s communication (Pontecorvo et al., 2024), but access to classes for caregivers is often delayed or limited (Lieberman et al., 2022). As a result, DHH individuals vary considerably in their early access to language, including exposure to mental state vocabulary (e.g., think, know, wonder; Milligan et al., 2007), which has been connected to later ToM development (Devine & Hughes, 2019)

In contrast, the 5–10% of DHH individuals who are born into DHH families typically experience different interactional dynamics. These families often employ visual and tactile strategies to engage with their children (Wille et al., 2019), leading to more successful and sustained interactions. Moreover, DHH families expose their DHH children to a common language where communication partners are more closely matched in their interaction styles since birth, which may support the early development of ToM components (Lederberg et al., 2013). Consequently, children from hearing families and children from DHH families may follow different developmental trajectories in ToM, and the literature documents substantial variability between these groups (Blose & Schenkel, 2022; Peterson & Siegal, 1999; Woolfe et al., 2002).

In summary, early parent–child interaction and access to language are variable both in terms of caregivers’ ability to make language accessible by learning sign, as well as the variability in the age at which DHH children receive cochlear implants and hearing aids. Such delays in access to spoken or sign language can negatively affect the quality of caregiver–child interactions, which are critical precursors to early ToM milestones (Baron-Cohen & Ring, 1994), and result in variable language and ToM development (Houston et al., 2012) which can hinder more advanced ToM components.

## 2. Research Questions of the Systematic Review

Do DHH individuals (Population), compared to a control sample of TH and/or among themselves (Comparator) in an assessment of ToM (Intervention), have differentiated results (Outcomes)?

We will review studies of ToM development and include factors of quality of interaction, early exposure to language (both sign and spoken), and age of access to hearing devices.

What ToM components have been studied in DHH individuals?

In wider research on TH individuals (Beaudoin et al., 2020), the ToM category beliefs hasbeen studied most often. We ask if this is the same for DHH individuals and if further research in other ToM areas is required.

## 3. Method

The authors conducted this systematic review in accordance with the guidelines for the Preferred Reporting Items for Systematic Reviews and Meta-Analyses (PRISMA) 2020 (Page et al., 2021). The research team agreed on and approved the protocol before it was uploaded to PROSPERO ref: CRD42024510783.

### 3.1. Eligibility Criteria

Table 1 shows the eligibility criteria and associated exclusion criteria for the searched studies. To reduce the subjectivity and differences between reviewers during the title/abstract and full text screening, the first and fourth authors applied the ‘Prioritization and Sequential Exclusion’ technique described by Saif-Ur-Rahman et al. (2022) and developed a priority protocol. This protocol detailed the exclusion criteria, which were ranked and ordered sequentially. In cases where a paper met multiple exclusion criteria, the authors assigned rejection based on the highest-priority criterion.

### 3.2. Information Sources and Search Strategy

The first author searched the following databases on 14 February 2024 and repeated the same process on 25 February 2025: ERIC, PsycINFO, PubMED, Scopus, Cochrane Databases, and Web of Science. To represent ToM and deaf concepts, using previous research, the following key terms were used and adapted to the title/abstract search method of each journal: (“theory of mind” OR “socioemotional” OR “social-pragmatic”) AND (“deaf*” OR “hearing impairment” OR “cochlear implant*” OR “hearing aids” OR “sign language”). No restrictions were made regarding the year of publication.

### 3.3. Selection and Data Collection Process

Search results were exported to Zotero 7.0, and duplicate papers were removed. The remaining articles were uploaded to Rayyan software in March 2024 (Ouzzani et al., 2016), when another duplicate search process was carried out. Authors L.M. and R.B.-C. used Rayyan software to blind screen the title/abstract content using the inclusion and exclusion criteria. The inter-rater agreement in this phase was 97% in 2024 and 100% in the 2025 search. Eligibility decisions were discussed and analysed in one meeting until raters reached full agreement. The first author, L.M., reviewed each eligible study for full text screening and extracted relevant data to answer the two research questions. The extraction form (Appendix B) was written by L.M. and reviewed by the other authors. All authors were involved in the process to gain consensus, check discrepancies, and make final decisions.

## 4. Results

The results of this review are divided into two parts. First, we provide a brief overview of the search results, the study characteristics, and their quality. Then, we present the findings following our research questions.

### 4.1. Search Results

**Figure 1:**
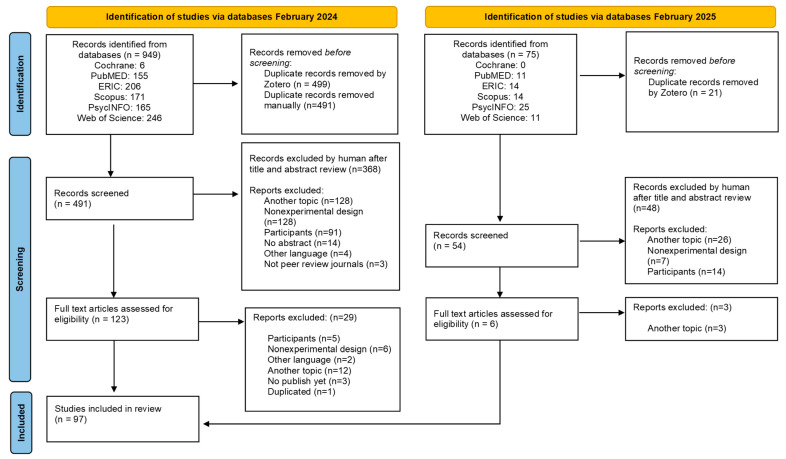
PRISMA flow diagram.

The selection process for included publications consisted of three phases: identification, screening, and inclusion (see Figure 1). In February 2024, author L.M. identified a total of 949 articles in the different databases. In this phase, Zotero software was used to remove duplicated papers; after that, 499 articles remained and were uploaded to Rayyan software. The software detected another 14 duplicated articles, and 7 articles were removed manually (n = 492). In the second phase, authors L.M. and R.B.-C. screened the title and abstract content using the inclusion and exclusion criteria in Table 1. If a paper met two exclusion criteria, reviewers applied the priority protocol to select the most important criterion. Disagreements regarding conflicts in the included or rejected articles and the chosen exclusion criterion, were discussed by L.M. and R.B.-C. After discussion, 123 papers were included for the next stage. Author L.M. screened papers for a full text review and rejected 29 articles that did not meet the inclusion criteria. Finally, 94 were included in the present study. Following this process, a second database search was conducted in February 2025 to ensure an updated review. The same phases were carried out by L.M.; first, 75 papers were identified by databases, then duplication removal was performed using Zotero (54), and title/abstract screening (6) and full text review followed; three more articles were included in the study.

An overview of descriptive information for the 91 studies is provided in Table A1 (Appendix B). The first study of ToM in DHH individuals was published in 1995. The last 10-year period of the review (2015–2025) was the most productive, with 48% (47/97) of the publications. The ages of the participants ranged from 1 year to 69 years. Only three studies included participants younger than three years old. Most of the studies (88%, 85/97) included preschool and school-aged children between 3 and 16 years old. The remaining studies utilized samples aged from 16 to 30 years old (8/97), with only one study including older individuals, aged 69 years. From the total studies, 25% (24/97) did not include a control group of TH but instead compared results from previous research (e.g., Macaulay & Ford, 2006, compared the performance of DHH children to the typical developmental pattern in which most TH children pass FB tasks between the age of 4 and 5 years). Regarding language, 42% (44/97) were conducted on English-speaking individuals (oral language or the related signed language, e.g., Australian Sign Language). The next most frequently used language, 9% (9/97), were in Spanish/Catalan individuals. All the studies used a quantitative design with just one longitudinal study. Overall, 79 of the 97 studies (75%) used a non-randomised case-control design. In addition to study design, studies employed a variety of methodological approaches. Instructions for the tasks were either explicit (using signs or spoken language), minimally verbal, or nonverbal. Participant responses were assessed using explicit measures in sign or spoken language, or through nonverbal responses (i.e., pointing, eye gaze, or looking-time patterns). We also noted variability in how the tasks were presented, with studies using videos, pictures, books, or real objects.

The first study documenting DHH participants’ use of hearing devices was published in 1999 (four years after the first DHH ToM publication). This study, carried out by Peterson and Siegal (1999), included a group of individuals with moderate to severe hearing loss who used hearing aids, but no further audiological details were provided. Until Jackson’s (2001) study of ToM in a group of cochlear implant users, the factor: hearing device, was not included again in the literature, even when participants with bilateral profound deafness (loss of more than 90 dB) and described as “predominantly oral” were included in research (Courtin, 2000). A lack of detailed participants’ audiological information was observed across many studies, e.g., an absence of aided hearing thresholds, scores on speech perception test (only six studies included this), unilateral or bilateral adaptation/implantation, or age of hearing access. Additionally, only 2 of the 97 studies, Panzeri et al. (2021) and Ziv et al. (2013), checked the hearing devices before testing to ensure that the child had optimal hearing for an oral task presentation. Continuing with participants’ information, some research, such as Chu (2025) or Choi and Jeong (2023), provided information about the type and duration of speech therapy received; however, that information is not reported in 97% (94/97) of the studies.

Research Question 1: Do DHH individuals (Population), compared to a control sample of TH and/or among themselves (Comparator) in an assessment of ToM (Intervention) have differentiated results (Outcomes)?

**Figure 2:**
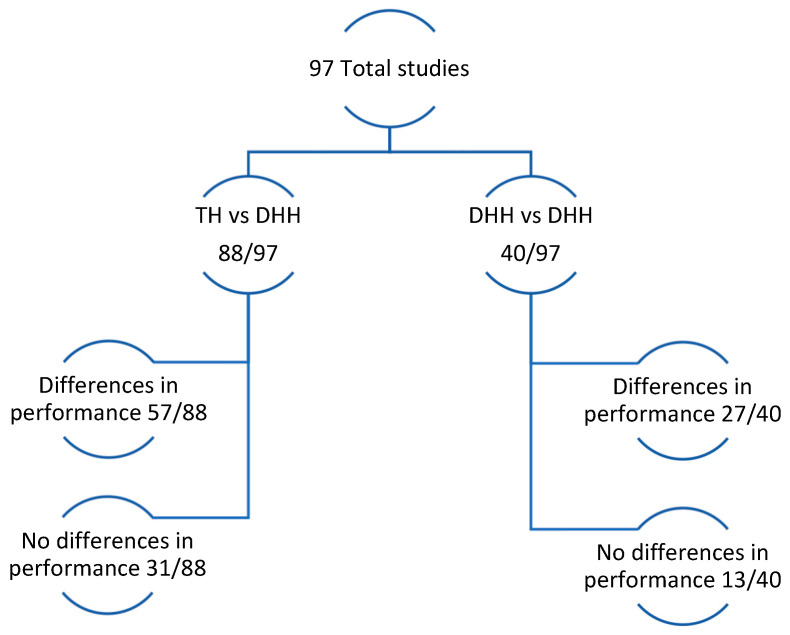
Overview of article comparison types and their findings.

Among the 97 papers included in this systematic review, some compared TH with DHH individuals, others compared different DHH subgroups, and several included both types of comparisons in the same study (Figure 2). The results for the current review are thus presented as comparisons between TH and DHH groups and between DHH subgroups.

First, we examined the comparisons between DHH and TH individuals, which were included in 91% (88/97) of the total papers. Results reported that 35% (31/88) of articles found no significant differences in performance or mixed results across different ToM components. Second-order FB and emotion recognition were the components with fewer differences between TH and DHH individuals across studies. It is noteworthy that the second-order FB studies revealed poor performance in both TH and DHH groups. Success in emotion recognition tasks was particularly high in DHH participants, with reports of no differences in comparison with the TH group in 60% (6/10) of the total studies assessing this component.

Considering the 88 papers that compared DHH and TH groups, 57 studies found TH individuals outperformed their DHH age-peers. Of the 31 studies reporting no differences, many papers attributed success to early language exposure. Ten studies examined this by including DHH individuals with DHH caregivers who used sign language and found that their ToM scores were comparable to those of age-matched TH individuals. The second approach, which analysed early language access, was used in 8 of the 31 studies, which compared early cochlear implant users with TH peers. Similarly, these eight studies found no significant differences in ToM performance between the groups. The remaining 13 papers that found no differences between DHH and TH groups, looked at individuals at different ages, different school environments (signing versus oral education), or did not identify a factor to explain the high performance of the DHH group.

Next, we examined studies (40/97) that looked at ToM performance between DHH sub-groups. Of those, 68% (27/40) revealed differences. Thirteen papers reported that DHH individuals from hearing families who had early language access—whether through early exposure to sign language or early hearing device use—performed better than those with late language exposure. In the remaining 14/27 papers, better performance of DHH individuals from DHH caregivers than those with hearing caregivers was reported. Having DHH caregivers generally implies early access to sign.

Research Question 2: What ToM components have been studied in DHH individuals?

**Figure 3:**
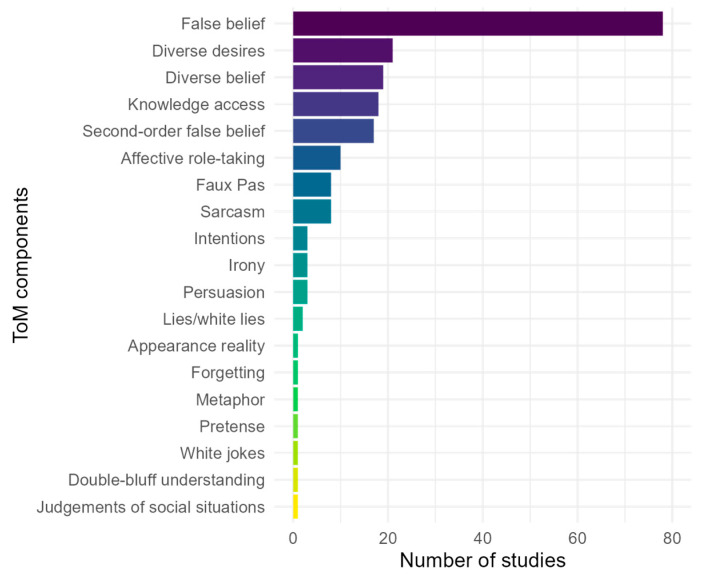
Most frequent ToM components in DHH studies (N = 91).

We found that 94% (91/97) of studies of ToM in DHH individuals assessed the construct by its different components, using single-task measurements. From those 91 studies, the component most studied was FB, which was assessed in 78 (86%) of articles. Figure 3 illustrates the distribution of the assessed components.

Moreover, 66% (58/88) of the studies explored ToM by assessing various developmental components (e.g., emotion recognition, diverse beliefs, diverse desires, false belief, second-order false belief, and moral reasoning; Meristo et al., 2007). The remaining studies analysed only one component (e.g., false belief; Aslıer et al., 2020).

The last 6% (6/97) of studies, assessed ToM as a global measure formed by a range of components using standardized ToM test batteries, e.g., The Theory of Mind Inventory-2 (Hutchins et al., 2017) or inventories such as the metacognitive tool (Al-Hilawani et al., 2002). Therefore, in these studies, results do not explicitly distinguish performance by specific ToM components but rather an overall multitask ToM score (Al-Hilawani et al., 2002; Blose & Schenkel, 2022).

### 4.2. Risk of Bias Assessment

The first author, L.M. conducted a quality assessment for all selected studies using The Joanna Briggs Institute Critical Appraisal Checklist for Case Control Studies (Moola et al., 2020). The checklist has nine criteria regarding quality, which determine the risk level of methodological bias (high, moderate, or low), such as clear and complete information about participants, criteria for identifying cases and controls, or the recognition of confounding factors, among others. The quality assessment of the 97 eligible studies resulted in 49% with a high risk, 30% with a moderate, and 21% with a low level of bias rating. Studies rated as having a higher risk of bias often lacked sufficient information on participant characteristics, did not include developmental assessment tests for comparison, or did not describe strategies to address potential confounding factors. We decided to include these studies despite the risk of bias for three reasons. First, the number of studies without bias is very low; secondly, the boundary between moderate and high risk is quite narrow and difficult to calculate for some papers. Lastly, research question 2 is not affected by methodological bias, as it is simply a review of the ToM tasks used in each paper. However, findings from studies deemed to have a high risk of bias should be interpreted with caution. The final score/decision for each study can be seen in Table A1.

## 5. Discussion

As far as we have ascertained, this is the first systematic review that focuses on DHH individuals and ToM. Performance across 97 studies compared different sub-groups of DHH individuals, as well as comparisons with TH individuals. There were also studies that used both types of comparisons in the same study. The aim of the SR was to synthesize the findings on ToM development in DHH individuals. We found that, from the studies that explored performance among DHH individuals compared to their TH peers, 65% (57/88) reported a delay in ToM for DHH individuals. On the other hand, from the articles that compared DHH subgroups, 68% (27/40) indicated differences within DHH individuals’ performance. Specifically, DHH individuals with DHH caregivers performed better than those with hearing caregivers, and individuals with early signed and spoken language access outperformed those with delayed access. Therefore, we conclude that while ToM development is delayed in some subgroups of DHH individuals, other subgroups do not show such delays. The variability across studies in ToM development among DHH individuals is associated with a range of factors, which we will discuss in relation to the relevant studies in the following sections. 

The review also examined how ToM has been assessed across its various components. We found that, of the 91 studies assessing ToM by component, 86% (78/91) focused on FB.

### 5.1. ToM Task Performance of DHH Individuals

The two most repeated factors used to explain differences in ToM development appearing in the reviewed papers were (1) the quality of early interactions, and (2) early access to language through both signed language and spoken language through the early use of hearing devices. Although these factors are often studied independently in the literature, they likely interact with each other, making both the analysis of their individual effects and their interaction complex. Despite the possible intersectionality (confluence of factors), we will discuss each factor in detail linked to the studies reviewed in our study.

#### 5.1.1. Quality of Early Interaction

In wider ToM developmental research, outside of deafness, considerable attention has been given to the role of family environment and parental involvement in supporting children’s ToM (Ensor & Hughes, 2008). Tomasello (2018) proposes that early social interactions with joint attention (e.g., following gaze, pointing, and sharing attention to objects) allow children to understand that others have perspectives different from their own, which serves as a precursor to understanding FB. Furthermore, Yu and Wellman (2023) found a positive correlation between interaction and ToM development. They reported that individuals who experienced a greater number and higher quality of social interactions showed higher ToM abilities.

A deafness diagnosis has a profound impact on these important precursors in the interaction skills of hearing families. It also influences parental emotional well-being and, consequently, the way hearing parents interact with their DHH children (Meadow-Orlans & Steinberg, 1993). Hearing caregivers generally use oral (auditory) strategies to communicate and struggle to attract their DHH child’s attention for long enough to maintain successful interactions and joint attention (Wille et al., 2019; Westby & Robinson, 2014). On the other hand, DHH caregivers tend to rely more on visual strategies, which result in longer instances of successful interactions with their DHH children (Wille et al., 2019). Consequently, these difficulties in establishing and maintaining effective interactions, specifically in hearing families, may limit DHH children’s opportunities for joint attention and rich social exchanges, ultimately hindering their ToM development.

Studies also describe DHH individuals with hearing caregivers as having reduced experience and participation in conversations early in life (Peterson & Siegal, 1995), reduced exposure to mental state language (Milligan et al., 2007), and less communicatively effective turn-taking (Morgan et al., 2014). Reduced early exposure to accessible ’mind-minded’ interaction, DHH individuals may miss out on the mental state language and conversational structures that play a crucial role for ToM development. This reduced early linguistic input may limit their understanding of others’ minds.

Returning to the papers in the review, many studies are relevant to each of these factors. Regarding interaction, Walker et al. (2017) found a bidirectional relation between early social interactions with both language and ToM development. Furthermore, Amraei et al. (2018) argue that the better ToM performance of DHH individuals from DHH in comparison to hearing caregivers is due to the use of visual strategies that can sustain communication from birth (eye contact, facial expression, and body language) and ensure children’s engagement in early conversations. Exploring additional results that highlight advantages for DHH individuals from DHH caregivers, Blose and Schenkel (2022) concluded that being raised by a DHH caregiver is a protective factor for ToM development, as this experience affords more opportunities for parents to engage in interactions with their DHH children using visual cues. Similarly, Falkman et al. (2007) explained that their results are in line with the essential factor of sharing a common language from early on, in order to establish quality communication and interaction between child and caregivers.

With respect to the content of interactions. Moeller and Schick (2006) found that hearing mothers produced less frequent mental state terms in their interaction with DHH children than mothers of TH children. They reported that DHH children who scored below 75% on a FB task were those whose mothers used fewer mental state terms and a smaller variety of different mental state words. Similarly, Lecciso et al. (2012) found hearing caregivers used significantly fewer mental state terms during interaction with their DHH children than caregivers with TH children, which they argue stems from caregivers’ perception of the complexity of topics their children can manage. Pluta et al. (2023) also identified qualitative differences in the conversational style of hearing caregivers interacting with DHH children compared to TH children. Caregivers of DHH children were more likely to refer to observable phenomena (physical characteristics) and less to mental state terms during shared book reading. In both studies, DHH individuals with hearing parents showed lower performance in ToM compared to their TH peers. It is interesting to note that Terwogt and Rieffe (2004) found that DHH children often failed to correct their hearing mother’s FB, in contrast to their TH peers. They suggested that this tendency to focus on their own desires may be due to communication barriers with their caregivers.

Another finding from the review on the specific topic of early interaction were the papers that identified good examples of interaction leading to age-appropriate ToM development. When comparing DHH individuals from DHH families to TH or DHH individuals from hearing families, many papers found that DHH individuals from DHH families exhibited similar ToM results to those of TH individuals (Lecciso et al., 2016; Meristo et al., 2007; Meristo & Strid, 2020; Schick et al., 2007; Woolfe et al., 2002). On the other hand, studies such as those by K. O’Reilly et al. (2014) and Jackson (2001) reported that DHH adults and children from DHH families outperformed those DHH individuals from hearing families. 

Overall, early quality interactions appear to play a crucial role in fostering ToM, providing a rich and natural setting for the development of communication and social cognition skills. In this context, a rich and natural setting refers to caregiver–child interactions that occur organically within the home and encompass behaviours such as joint attention, turn-taking, connectedness, and the use of emotional and mental state language (Ensor & Hughes, 2008). The results further suggest that DHH families tend to engage in more effective interactions with their DHH children, which may, in turn, support their ToM development. However, it is noteworthy that optimal interactions between hearing mothers and their DHH children have also been reported when mothers receive additional support from various sources, such as family members and early intervention professionals of social support services (Meadow-Orlans & Steinberg, 1993).

In conclusion, an explanation for ToM development coming from early interaction (Ensor & Hughes, 2008) is borne out of our review of the literature. Difficulties and successes in establishing attention and providing quality interactions lead to variable results on ToM development.

#### 5.1.2. Early Access to Language

The second major factor highlighted across papers in this review, as a potential explanation for ToM development in DHH individuals, is the difference between groups in their exposure to early accessible language. The broader literature on ToM development emphasizes several linguistic factors, including caregivers’ everyday use of mental-state talk (Devine & Hughes, 2019; Ruffman et al., 2002), a diverse vocabulary (Farrar & Maag, 2002), and specific sentence-level structures (J. G. de Villiers & de Villiers, 2014; Milligan et al., 2007).

Concerning DHH individuals exposed to early sign language by their DHH caregivers, studies indicate significant advances in ToM development, with some reporting performance comparable to that of TH peers (Courtin & Melot, 2005; Lecciso et al., 2016; Peterson et al., 2016b; Tomasuolo et al., 2012). However, the review found that the use of signed language alone in hearing families was insufficient to support robust ToM development; it also required caregivers to understand their child’s needs beyond language. The reviewed studies on DHH individuals from hearing families who learnt and used sign language have mixed results for ToM development (Falkman et al., 2007; Hao et al., 2010; Siriattakul et al., 2021). While hearing caregivers could learn and use signed language at intermediate levels (Pontecorvo et al., 2024), their proficiency appears insufficient to foster ToM development in the same way it does for DHH caregivers. Several studies concurred on this point. For example, Falkman et al. (2007) found no significant correlation between the quality of hearing care givers’ sign use and their DHH children’s results on the ToM tasks. Furthermore, Edwards et al. (2021) found that DHH adults who rated their sign language skills as higher demonstrated a poorer understanding of sarcasm and metaphors compared to DHH adults with stronger spoken language skills.

The role of language in ToM development can also be seen in studies that have looked at the early use of hearing devices. The term ‘early’ varies across studies but can be considered sometime before 24–36 months in the fitting of cochlear implants. Both Ziv et al. (2013) and Figueroa et al. (2020) found comparable ToM performance between DHH children with early cochlear implants who used spoken language and their TH peers. Similarly, Sundqvist et al. (2014) found no significant differences in emotional ToM performance between early-implanted DHH children and TH children. Furthermore, Panzeri et al. (2021) and Choi and Jeong (2023) reported no significant differences in performance between DHH children with early hearing device use and their TH peers matched for hearing age and receptive language abilities.

Early use of hearing devices means that DHH children have more time and exposure to mind related labels (Devine & Hughes, 2019; Ruffman et al., 2002; Farrar & Maag, 2002) and sentence structures (J. G. de Villiers & de Villiers, 2014; Milligan et al., 2007) that have been linked to ToM development. Comparisons across groups remain challenging because most DHH individuals are exposed to varying degrees of both spoken language and sign language, rather than exclusively acquiring one modality. Lundy (2002) and Peterson (2004) reported similar ToM outcomes between late-signing children and those relying solely on oral communication with hearing devices, with both groups showing poorer performance compared to their TH peers. However, these studies did not specify whether participants were early or late users of hearing devices, nor did they provide other relevant audiological information, which complicates the comparison. In this context, Panzeri et al. (2021) emphasized the influence of prosody and intonation in tasks involving second-order false belief and the understanding of irony, highlighting the critical role of adequate auditory access. To better understand factors affecting ToM performance, detailed audiological data—such as aided hearing thresholds and speech perception abilities—are essential. Several studies included in the systematic review underscore the importance of reporting these audiological variables when evaluating ToM development (Delkhah et al., 2021; Marschark et al., 2019).

In summary, the studies reviewed highlight that ToM development is supported by the interacting factors of early quality interaction, parents who are able to use an accessible language with a high quantity of mental state vocabulary, as well as, the benefits of the early use of a hearing device.

#### 5.1.3. Other Variables

Previous research on DHH individuals raised other factors that were linked to variability in both general and ToM development, including the type and duration of speech therapy (Holzinger et al., 2020), early hearing detection and intervention (Yoshinaga-Itano et al., 2020), and family structure (Johnson et al., 2025). These factors interplay in shaping the interactions and the overall development of DHH individuals. In the majority of papers included in this systematic review, information about these variables was mostly absent from the participant demographics. Additionally, the quality of the methodologies for a significant proportion of included studies does not meet optimal standards. This heterogeneity in quality poses a challenge for comparing results and replicating the research. Overall, ToM development in DHH individuals is complex, with group differences likely stemming from the interplay of multiple factors. The lack of comprehensive data, particularly regarding audiological profiles, speech therapy history, and caregivers’ involvement, limits the ability to fully interpret findings.

### 5.2. ToM Components Assessed in DHH Individuals

The second research question concerned the components studied in DHH individuals’ ToM development. It was confirmed that not all areas of ToM have been studied to the same extent in DDH individuals. Beaudoin et al. (2020) looked at TH children and found that the most studied category was beliefs, which includes FB and second-order FB, and the area that received the least attention was intentions. In the case of DHH individuals, the same pattern was observed.

As described in the introduction, FB tasks are over-used in TH and DHH individuals because of the particular demands they entail. By the time the earliest DHH paper identified in this review was published (Peterson & Siegal, 1995), a considerable amount of research on FB in TH individuals (Wimmer & Perner, 1983), and individuals with autism (Baron-Cohen et al., 1985), had already been established in the preceding decade. Peterson and Siegal (1995) explain that the decision to use FB tasks with DHH individuals was influenced by its extensive application in previous research with individuals with autism and other disabilities (e.g., Down syndrome). This prior research allowed Peterson and Siegal (1995) to compare the DHH sample’s performance with previous data from individuals of different ages and abilities on a standardized task. Furthermore, FB tasks are optimal for assessing DHH individuals because of the following characteristics: FB tasks use a simple story line with simple vocabulary to understand, use nonverbal responses, and use visually salient prompts, such as puppet play, facilitating the comprehension of the narrative (Peterson & Siegal, 1995; Steeds et al., 1997). These factors enabled replication of FB tasks with similar protocols, and as extension, the testing of individuals with different language abilities. Furthermore, ToM assessments may be adjusted to minimize the impact of executive functions and language skills on task performance. For instance, Pluta et al. (2021) modified traditional FB tasks by presenting them in the form of short cartoons, without introducing an elaborate storyline, and participants responded by touching a screen non-verbally. Additionally, Pyers and Senghas (2009) developed a low-verbal picture-completion task, which improved the understanding of FB in a sample of DHH adult Nicaraguan late signers.

Additionally, the typical age range for FB tasks, from 4 to 5 years, corresponds with the preschool and early childhood years, which offer practical advantages for participant recruitment (e.g., Ketelaar et al., 2012; Meristo et al., 2012). Children of this age can be accessed more easily through schools, which provide centralized information. Conversely, testing FB in DHH infants, younger than 3 years is more challenging. In the current systematic review only 3% (3/97) of articles included DHH participants younger than 3 years old. Younger children are recruited through various sources with potentially more barriers such as hospitals, audiology centres, or early intervention programs. Nonetheless, recruiting younger children remains crucial to understand how DHH infants comprehend intentions and use this understanding to build more advanced ToM elements (Baldwin, 2000).

### 5.3. Limitations of the Review Process

There are some issues that might affect the results of this study. Only papers published in peer-reviewed journals, in English and Spanish, were selected, potentially missing some information from the grey literature or written in other languages. However, this criterion, of peer-reviewed journals, is a guarantee of quality and scientific rigor. During the search process, it was necessary to use various keywords focusing on ToM to capture all the papers included. It is possible that more studies could have met the inclusion criteria but were not included because of the heterogeneous terms used for the ToM aspect.

Across studies, we observed mixed results, which might be linked to differing methodologies. Jones et al. (2015) found no differences in performance between TH and DHH individuals on both unexpected-location and unexpected-content FB tasks. They attributed this finding to the methodology used, in which a native signer (not an interpreter) presented the task, maximizing the clarity of the task requirement for individuals by reducing the attentional shift demands. In the same way, Courtin (2000) argued that differences in performance of DHH with early and late sign language exposure could have stemmed from the presence of two adults during the testing of late signers, whereas early signers were tested in the presence of only one adult, i.e., a more direct evaluation. Additionally, it is important to verify the language of testing. In the case of DHH individuals who communicate using spoken language by hearing, only two studies checked the correct working of hearing devices before testing, and just six ensured adequate aided speech perception.

Finally, the scoring of ToM tasks varies significantly across studies. Sobel and Austerweil (2016) explored the impact of different coding methods in FB research, e.g., excluding children who failed the control measure (Perner et al., 1987), and concluded that these variations can lead to misleading conclusions about children’s ToM outcomes rather than true developmental differences. An inconsistency in the coding schemes for “pass/no pass” on tasks may complicate the process of comparing results across studies. All of these methodological issues may play a role in the final ToM results.

### 5.4. Conclusions and Clinical Implications

This systematic review highlights the complex interplay of factors influencing ToM development in DHH individuals, including the quality of social interaction and early exposure to both sign and spoken language. These results obviously raise the importance of early intervention, particularly promoting rich communication environments immediately after diagnosis, to foster ToM development. The review also reveals a critical gap in research, particularly in exploring components beyond FB such as intentions and second order FB. Future studies should also provide more complete information about DHH participants so as to link these factors more clearly to variability in development of ToM.

## Figures and Tables

**Table 1:** Eligibility criteria and priority exclusion.

Inclusion Criteria	Exclusion Criteria	Priority
Target population: DHH prelingual individuals with no developmental comorbidity. Any or no amplification Any modality of communication	Participants: No DHH participants in the study Presence of developmental comorbidity	7
Papers measuring ToM performance during ToM assessment	Another topic: Topics not concerned to the main question. Experiment does not assess ToM or participants ToM task performance Papers that assess cognitive skills or social skills possibly related to ToM but not ToM abilities	6
Only papers published in peer-reviewed journals	Not peer-reviewed articles No abstract available Duplication	4 1 2
Papers with experimental research, quantitative results. Cross-sectional studies, between-groups design, and longitudinal studies	Nonexperimental design: Opinion papers, case studies, literature or systematic review. Treatment/Training studies will be included if pre-treatment/training TOM results are included	5
Papers written in English or Spanish	Languages other than English or Spanish	3

## Data Availability

All data supporting the reported results are included within the article in the form of tables. No additional datasets were generated or analyzed during this study.

## Appendix A

### Data Extraction

General

Authors
  Publication year

Participants

Sample size of typical hearing individuals
  Sample size of typical deaf and hard-of-hearing individuals
  Type of hearing devices used and bilateral/unilateral
  Age of hearing Access
  Communication modality and language
  Age interval

Study

Risk of Bias
  ToM component assessed and performance
  ToM task
  Main results
  Results from comparing language modality
  Results from comparing hearing devices
  Results from comparing family hearing status
  Task presentation
  Other conclusions
  Executive functions assessment
  Language assessment
  Communication/interaction assessment
  IQ assessment
  Other measures

## Appendix B

**Table A1:** Studies included in the systematic review (are labelled with * Asterisks in the References).

Authors	Year	TH	DHH	HD	Family Hearing Status	Age Interval (Years)	ToM Component and Performance	TH vs. DHH	DHH vs. DHH	Quality Papers
(Peterson & Siegal, 1995)	1995	0	26	NR	25 HF 1 DF	8.1;13	False belief	TH > DHH	-	***
(Steeds et al., 1997)	1997	0	22	NR	HF	5.8;12.4	Diverse desires False belief	TH > DHH	-	***
(Peterson & Siegal, 1998)	1998	Ex1—35 Ex2—47	Ex1—30 Ex2—24	NR	HF	DHH-5.9;11.10 TH-3.6;4.5 DHH-6.2;12.5 TH-3.6;4.5	False belief	TH > DHH	-	***
(Russell et al., 1998)	1998	0	32	NR	30 HF 2 DF	DHH1-4.9;7.9 DHH2-8.9;12.6 DHH3-13.6;16.11	False belief	TH > DHH	-	***
(Peterson & Siegal, 1999)	1999	21	59	NR	48 HF 11 DF	DHH-5.6;13.2 TH-3.11;4.4	*False belief*	TH = DoDP & DoHP(oral)	DoHP(oral) & DoDP > DoHP(SL)	**
(Courtin, 2000)	2000	39	155	NR	99 HF 37 DF	DHH-5;8 TH-4;6	*False belief*	TH = DoDP	DoDP > DoHP	***
(Marschark et al., 2000)	2000	15	15	NR	HF	DHH-9.7;15.10 TH-10.6;15.5	**False belief (Mental state attribution)**	TH > DHH	-	***
(Rhys-Jones & Ellis, 2000)	2000	40	34	NR	28 HF 6 DF	6;11	*Comprehension and judgments of social situations*	TH > DHH TH (11;16y) = DHH(11;16y)	-	**
(Rieffe & Terwogt, 2000)	2000	85	23	NR	22 HF 1 DF	6;10	**Emotion recognition**	TH = DHH	-	***
(Figueras-Costa & Harris, 2001)	2001	0	21	1 CI—NR 20 HA—NR	HF	DHH1-4.5;6.4 DHH2-6.7;11.9	False belief	TH > DHH	-	**
(Jackson, 2001)	2001	48	50	5 CI—NR	46 HF 4 Dfamily	Early SL-5.2;10.10 Late SL-4.10;8.3 SL & Oral-5;12.11 Oral-4.11;11.11	False belief **2nd-order false belief**	TH > DHH	Dfamily > Hfamily EarlyS L > LateSL & oral	***
(Scott et al., 1999)	1999	0	22	NR	20 HF 2 DF	DHH1-5.1;8.3 DHH2-9.1;12.10	Diverse desires Diverse intentions	TH > DHH	-	***
(Al-Hilawani et al., 2002)	2002	44	28	NR	NR	8;11	**Cognitive ToM**	TH = DHH	-	***
(Lundy, 2002)	2002	0	29	9 CI—NR 20 HA—NR	HF	5;10	False belief	TH > DHH	DHH(oral) = DHH(SL)	***
(Peterson, 2002) Ex1 Ex2	2002	25 26	21 21	NR	HF	DHH-6.8;12.6 TH-4.1;5.8 DHH-6.11;12.11 TH-3.10;5.1	False belief	TH > DHH	-	***
(Woolfe et al., 2002) Ex1 Ex2	2002	40 0	51 39	NR	32 HF 19 DF 21 HF 18 DF	DHH-4;8 TH-3;4	False belief	-	DoDP > DoHP	***
(Rieffe et al., 2003)	2003	67	53	NR	52 HF 1 DF	DHH-9.2;11.8 TH-9;11.2	Emotion recognition	TH > DHH	-	***
(Woolfe et al., 2003)	2003	0	20 + 20 siblings	NR	7 Hsibling 13 Dsibling	Hsibling-4;8.6 Dsibling-4.2;16.3	False belief	-	Dsibling = Hsigling	*
(Howley & Howe, 2004) Ex1 Ex2	2004	10 20	10 25	NR 21 HA—NR	4 HF 6 DF 20 HF 5 DF	6.94;8.93 DHH1-5.08;8.42 DHH2-8.42;11.58	Affective role-taking **Perceptual role-taking**	TH > DHH TH = DHH	DoHP = DoDP	***
(Peterson, 2004)	2004	17	26	13 CI—NR 13 HA—NR	HF	4;12	False belief	TH > DHH	DHH(bl school) = DHH(oral school)	***
(Terwogt & Rieffe, 2004)	2004	36	21	NR	20 HF 1 DF	DHH-10.5;12.4 TH-9.8;12.10	Diverse desires False belief	TH > DHH	-	**
(Courtin & Melot, 2005)	2005	36	88	NR (CI excluded)	60 HF 28 DF	5;7	Appearance-Reality *False belief*	TH > DHH DoDP >TH	DoDP > DoHP	***
(Peterson et al., 2005)	2005	62	47	NR	36 HF 11 DF	3.7;13.7	**Diverse beliefs** **Diverse desires** **Knowledge access** **Hidden emotion** *False Belief*	TH = DHH TH > DoHP	DoDP > DoHP	**
(Falkman & Hjelmquist, 2007)	2007	10	10	4 CI—NR	8 HF 2 DF (no SL)	7.4;11.3	False belief	TH > DHH	-	*
(A. M. González-Cuenca & Quintana García, 2006)	2006	0	54	NR	HF	6;19	False belief	TH > DHH	-	***
(Macaulay & Ford, 2006)	2006	0	10	10 CI—NR	HF	DHH-4.4;11.1	False belief	TH > DHH	EarlyCI = LateCI	***
(Moeller & Schick, 2006)	2006	26	22	10 CI—NR 10 HA—BI	HF	DHH-4.25;9.92 TH-4.25;5.92	False belief (verbal) **False belief (Nonverbal)**	TH > DHH TH = DHH	-	*
(Morgan & Kegl, 2006)	2006	0	22	NR	HF	Early SL-8;24 Late SL-12;39	False belief	-	EarlySL > LateSL	*
(Peterson & Slaughter, 2006) Ex1 Ex2	2006	13 17	21 17	NR	HF	6;11 DHH-6;12 TH-4;5	Knowledge access False belief	TH > DHH	-	**
(Falkman et al., 2007)	2007	10	10	3 CI—NR	HF	DHH-7;9.6 TH-8;9.6	Diverse belief False belief	TH > DHH	Wide variability	***
(A. M. González-Cuenca et al., 2007)	2007	0	54	No CI	HF	6;19 14;19	False belief	TH > DHH	-	***
(Meristo et al., 2007) Ex1 Ex2	2007	105 26	97 61	NR	41 HF 56 DF 37 HF 24 DF	Late SL-6.5;12.9 Native SL-4.6;12.8 TH-3.5;10.8 7.4;16.1	*True belief* *False belief* *Emotion Recognition* *Diverse beliefs* *Diverse desires* *False belief* *2nd-order false belief* *Moral reasoning*	TH = DoDP(bl school)	DoDP(bl school) > DoDP & DoHP(bl school)	***
(Schick et al., 2007)	2007	42	176	33 CI—NR 53 HA—NR	127 HF 49 DF	4;8.38	*False belief* *FB Verbal* *FB Nonverbal* *Knowledge access*	TH = DoDP	DoDP > DoHP	*
(A. M. González-Cuenca et al., 2008)	2008	0	54	No CI	HF	6;19	False belief 2nd-order false belief	TH > DHH	-	***
(Meristo & Hjelmquist, 2009)	2009	0	61	NR	37 HF 24 DF	7.4;16.1	Emotion Recognition Diverse beliefs Diverse desires False belief 2nd-order false belief Moral reasoning	-	EarlySL(bl school) > EarlySL(oral school) & LateSL(blschool)	***
(Peterson & Wellman, 2009)	2009	60	33	12 CI—NR	HF	DHH-5.10;13.6 TH-2.8;5.9	Diverse beliefs Diverse desires False belief Knowledge access Hidden emotion Social pretending	TH > DHH	-	**
(Peters et al., 2009)	2009	0	30	30 CI—NR	HF	3.1;12.0	False belief	-	-	*
(Pyers & Senghas, 2009)	2009	0	18	NR	NR	EarlySL-26.8;28.7 LateSL-17.5;19.1	False belief	-	EarlySL > LateSL	***
(Hao et al., 2010)	2010	32	53	NR	45 HF 8 DF	21.5 mean age	*Advanced ToM implicit* Advanced ToM explicit	TH = DoDP TH > DHH	DoDP > DoHP	**
(Torres & Rodríguez, 2011)	2011	25	25	10 CI—NR 15 HA—NR	HF	DHH-4.17;16.5 TH-4.5;16.33	Graphic **False belief graphic** **2nd-order false belief** Standard False belief 2nd-order false belief	TH = DHH TH > DHH	-	***
(Wellman et al., 2011)	2011	61	31	19 CI—NR	HF	Chinese-3.1;3.11 English-3.1;5 Auslan-4.2;12.8	Diverse desires Diverse beliefs Knowledge access False belief Hidden emotion	TH > DHH	-	***
(P. A. de Villiers & de Villiers, 2012)	2012	45	45	25 CI—NR 20 HA—NR	HF	DHH-4.6;7.11 TH-3.1;5.11	False belief	TH > DHH	-	***
(Ketelaar et al., 2012)	2012	69	72	72 CIs 2/3 CI—UNI 1/3 CI—BI	HF	1;6	**Intention** **Common-desire** Uncommon-desire False belief	TH = DHH TH > DHH	-	**
(Lecciso et al., 2012)	2012	17	17	17 HA—NR	HF	5.4;4.6	False belief 2nd-order false belief Mentalistic understanding of non-literal communication Perceptual component of ToM	TH > DHH	-	**
(Levrez et al., 2012)	2012	12	12	5 CI—NR 7 HA—NR	HF	DHH-9.3;12.2 TH-6.7;8.3	False belief	TH > DHH	-	**
(Meristo et al., 2012)	2012	10	10	5 CI—NR 5 HA—NR	HF	DHH-1.5;2.2 TH-1.7;2.3	False belief **True belief**	TH > DHH TH = DHH	-	**
(Peterson et al., 2012)	2012	68	31	NR	HF	3;13	Diverse beliefs Diverse desires False belief Hidden emotion Knowledge access Sarcasm Understanding	TH > DHH	-	***
(Tomasuolo et al., 2012)	2012	15	30	22 HA	23 HF 7 DF	DHH-6.1;14.6 TH-6.6;13.9	*False belief*	DHH(bl school) > TH TH > DHH(SL school)	DHH(blschool) > DHH(SLschool)	***
(Macaulay & Ford, 2013)	2013	47	48	21 CI—NR 27 HA—BI	46 HF 2 DF	DHH-4;11 TH-3;5	False belief 2nd-order false belief	TH > DHH	-	**
(Ziv et al., 2013)	2013	23	30	22 CI—UNI 8 HA—BI	20 HF 10 DF	5;7	*Emotion recognition* *False belief*	TH = DHH(oral) TH > DHH(SL)	DHHCI > DHHNoCI	*
(Hao & Su, 2014)	2014	25	22	NR	HF	DHH-9;13.11 TH-4;6.11	**Emotion recognition** False belief no eye-gaze cues **False belief eye-gaze cues**	TH = DHH TH > DHH TH = DHH	-	**
(K. O’Reilly et al., 2014) Ex1 Ex2	2014	39 35	42 35	17 CI—NR	32 HF 10 DF 27 HF 8 DF	5;12 18;69	*False belief* *2nd-order false belief* *Sarcasm*	TH > DHH TH(old) = DoDP(old) TH(old) > DoHP(old)	DoDP > DoHP	**
(Sundqvist et al., 2014)	2014	18	16	14 CI—BI 2 CI—UNI	HF	DHH-4.25;9.5 TH-6;8	*Emotion recognition* False belief 2nd-order false belief	TH = EarlyCI TH > LateCI TH > DHH	EarlyCI > LateCI	**
(Jones et al., 2015)	2015	46	27	14 CI—NR 13 HA—NR	HF 12—signers 15—No signers	DHH-6.7;12.1 TH1-4.5;5.9 TH2-6.1;11.6	**False belief (location)** *False belief (content)* 2nd-order false belief	TH1 = DHH TH2 > DHH	DHH(oral) > DHH(SL)	**
(Peterson, 2016) Ex1 Ex2	2016	31 21	30 35	12 CI—NR 18 CI—NR	HF	DHH-5.92;12.92 TH-4.25;10.50 DHH-5.67;13.33 TH-7.33;12.42	Diverse beliefs Diverse desires Knowledge access False belief Hidden emotion Sarcasm Understanding	TH > DHH	-	***
(Holmer et al., 2016)	2016	0	13	Unclear	11 HF 2 DF	7.3;14.10	Diverse desires Diverse beliefs Knowledge access False belief Hidden emotion	TH > DHH	DoHP = DoDP	**
(Lecciso et al., 2016)	2016	47	47	4 CI—NR 19 HA—NR	32 HF 15 DF	15.9;28.1	*2nd-order false belief* *Advanced ToM (misunderstanding of intention, persuasion, white lies, double buff)* Mentalistic understanding of non-literal communication	TH = EarlySL TH > DHH(oral & LateSL) TH > DHH(EarlySL, LateSL &oral)	DoDP > DoHP	**
(Meristo et al., 2016)	2016	15	15	5 CI—BI 3 CI—UNI 7 HA—NR	HF	DHH-CI-3.11;8.5 DHH-HA-4;7.11 TH-4.1;8.11	False belief spontaneous *False belief elicited*	TH > DHH TH = DHH CI TH > DHH HA	-	**
(Peterson et al., 2016a)	2016	21	36	18 CI—NR	HF	DHH-5.67;14.7 TH-7.33; 13.42	Diverse beliefs Diverse desires Knowledge access False belief Hidden emotion Sarcasm Understanding	TH > DHH	-	***
(Peterson et al., 2016b)	2016	53	66	29 CI—NR	54 HF 12 DF	DHH-5.00; 12.08 TH-6.00; 13.42	*Diverse beliefs* *Diverse desires* *knowledge access* *False belief* *Hidden emotion*	TH = DoDP TH > DoHP	DoDP > DoHP	***
(Fujino et al., 2017)	2017	0	369	NR	NR	4;12	False belief	TH > DHH	-	***
(Hutchins et al., 2017)	2017	0	12	11 CI—NR 1 HA—NR	HF	5.2;11.1	Early, basic, and advanced skills	TH > DHH	EarlyCI > LateCI	*
(Serra et al., 2017)	2017	30	10	NR	NR	DHH-4.5;9 TH-4.5;6	Emotion recognition **Emotion recognition (visual cues)**	TH = DHH TH > DHH	-	***
(Walker et al., 2017)	2017	106	272	0 CI 134 HA—NR	HF	5;6	False belief **2nd-order False belief**	TH > DHH TH = DHH	-	*
(Amraei et al., 2018)	2018	0	30	30 CI—NR	15 HF 15 DF	HF-5;8 DF-3;8	False belief	-	DoDP > DoHP	***
(Liu et al., 2018)	2018	46	36	36 CI—NR	HF	3.8;6.9	Diverse desire False belief	TH > DHH	-	**
(Marschark et al., 2019)	2019	41	94	46 CI—UNI	81 HF 9 DF	NR; college students	2nd-order false belief Double-bluff understanding Sarcasm	TH > DHH	DoHP = DoDP	**
(Peterson & Wellman, 2018a)	2018	37	27	NR	HF	3;11	Diverse beliefs Diverse desires Knowledge access False belief Hidden emotion Sarcasm Understanding	TH > DHH	-	***
(Peterson et al., 2018) Ex1	2018	43	28	NR	HF	DHH-5.25;11.17 TH1-7.25;10.17 TH2-3.83;5.00	False belief Diverse beliefs Diverse desires Knowledge access False belief Hidden emotion Persuasion Sarcasm	TH > DHH	-	***
(Peterson & Wellman, 2018b)	2018	44	31	17 CI—NR	HF	DHH-5.83;12.42 TH-3.08;5.00	**False belief (prediction)** False belief (explanation)	TH = DHH TH > DHH	-	**
(Smogorzewska et al., 2018)	2018	243	268	74 CI—NR 145 HA—NR	HF	DHH-5.11;10.11 TH-5.11;12.0	Diverse desires Diverse beliefs False belief Knowledge access Content false belief Hidden emotion Faux pas	TH > DHH	-	***
(Aslıer et al., 2020)	2020	82	77	77 CI—UNI	HF	3;9	False belief	TH > DHH	EarlyHD > LateHD	*
(de Gracia et al., 2020) Ex1 Ex2	2020	150 0	59 42	NR NR	HF 41 HF 1 DF	DHH-8;14.58 TH1-8.75;14.83 TH2-3.33; 7.92 DHH-15.25;22.17 TH1-8.75;14.83 TH2-3.33;7.92	Diverse desires Diverse beliefs Knowledge access Content false belief Hidden emotion	TH > DHH	-	**
(Edwards et al., 2021)	2021	38	74	26 CI—NR 6 CI + HA	NR	18;24	Sarcasm Metaphor Inference making Tasks	TH > DHH	-	*
(Figueroa et al., 2020)	2020	54	36	3 HA + CI 10 CI—BI 23 CI—UNI	HF	DHH-14.03;21 TH-13.5;18	**Affective ToM: False belief** *Cognitive ToM: False belief*	TH = DHH TH > DHH	EarlyCI > LateCI BICI > UNICI	*
(A. González-Cuenca & Linero, 2020)	2020	38	58	20 CI-UNI	HF	10;19	False belief 2nd-order belief Selfish and white lies Critical irony	TH > DHH	DHHCI = DHHNoCI	***
(Meristo & Strid, 2020)	2020	24	22	7 CI—BI 6 CI—UNI	22 HF 24 DF	1.5;8.9	*False belief*	TH = DoDP TH > DoHP	DoDP > DoHP	***
(Sidera et al., 2020)	2020	75	54	22 CI—NR 30 HA—NR	8 DF 24 Drelative	DHH-3.4;8.9 TH-3.2;8.9	Emotion recognition (understanding pretend emotions)	TH > DHH	DHHCI = DHHHA	**
(Yu et al., 2021)	2020	0	84	12 CI—UNI 16 CI—BI 56 HA—BI	HF	3;6	Diverse desires Diverse beliefs Social pretend Knowledge access False belief	-	EarlyHD > LateHD	*
(Delkhah et al., 2021)	2021	36	36	36 CI—NR	NR	5;9	Basic ToM skills Advanced ToM skills	TH > DHH	-	**
(Panzeri et al., 2021)	2021	56	28	20 CI—BI 8 CI + HA	HF	DHH-4;12 TH1-5;12 TH2-3;10	Emotion recognition *False belief* *2nd-order false belief* Irony	TH > DHH TH1 > DHH TH2 = DHH TH > DHH	-	*
(Pluta et al., 2021)	2021	94	45	29 CI—BI 7 CI—UNI 9 CI + HA	HF	DHH-3.25;8.25 TH-3.25;7.25	*False belief*	TH(4;5y) > DHH(4;5y) TH(6;8) = DHH(6;8)	-	*
(Siriattakul et al., 2021)	2021	0	200	NR	190 HF 10 DF	8;15	False belief	TH > DHH	DoDP > DoHP	***
(Smogorzewska & Osterhaus, 2021)	2021	237	234	Unclear	NR	7.5;9.5	2nd-order false belief Advanced ToM: Faux pas	TH > DHH	-	***
(Blose & Schenkel, 2022)	2022	144	104	23 CI—NR	13 DF 24 Dsibling 67 Dfamily	21 mean age	*Affective ToM*	TH > DoHP	DoDP > DoHP	***
(Durrleman et al., 2022)	2022	0	21	9 CI—BI 2 CI—UNI 5 CI—HA 5 HA—NR	HF	5.9;11	False belief	TH > DHH	-	***
(Figueroa et al., 2022)	2022	0	8	4 CI—BI 4 CI—UNI	HF	10;14	Diverse desires Diverse beliefs Knowledge access Content false belief Hidden emotion Cognitive ToM: False belief Affective ToM: Faux pas	-	EarlyCI > LateCI	*
(Smit et al., 2022)	2022	34	14	NR	11 HF 3 DF	NR	Lies White joke Pretense Persuasion Appearance/reality misunderstanding Forgetting Double bluff Contrary emotions Irony	TH > DHH	-	***
(Akkaya & Doğan, 2023)	2023	100	100	33 HA—NR 68 CI—NR	NR	4;5.9	**Emotion recognition** *False belief*	TH = DHH TH (5y) = DHH (5y) TH (5.5y) > DHH (5.5y)	EarlyHD > LateHD	**
(Choi & Jeong, 2023)	2023	50	50	11 HA + CI 39 CI—BI	HF	2;12	**Early skills** **Basic skills** Advanced skills	TH = DHH TH > DHH	-	*
(Pluta et al., 2023)	2023	52	39	39 CI—BI	HF	DHH-2.9;7.8 TH-3;5.8	Early ToM Basic ToM Advanced ToM Combined ToM	TH > DHH	-	***
(Tuohimaa et al., 2023)	2023	45	86	22 CI—BI 19 HA—BI	HF	4;4.9	Contextual Inference with/without ToM demand: (Include feeling recognition and false belief)	TH > DHH	EarlyHD = LateHD	*
(Meristo et al., 2024)	2024	18	12	8 CI—BI 5 CI—UNI	HF	3.9;8.4	**False belief (verbal)** False belief (Nonverbal)	TH = DHH TH > DHH	-	**
(Remmel & Peters, 2009)	2009	30	30	29 CI—UNI 1 CI—BI	HF	DHH-3.1;12 TH-4.5;6.4	**Diverse desires** **Diverse beliefs** **Knowledge access** **False belief** **Hidden emotion** **Nonverbal False belief** **Real apparent Emotion**	TH = DHH	EarlyCI > LateCI	*
(Serrat et al., 2024)	2024	25	22	7 CI—BI 2 CI—UNI 2 CI + HA 11 HA—BI	HF	DHH-8;12 TH-8;12	False belief 2nd-order false belief	TH > DHH	-	**
(Tuohimaa et al., 2025)	2025	65	54	29 CI—BI 25 HA—BI	HF	6.0;6.11	Contextual Inference with/without ToM demand: (Include feeling recognition and false belief)	TH > DHH	DHHCI = DHHHA	*
(Chu, 2025)	2025	53	57	31 CI—NR 26 HA—NR	HF	4.6;5.75	**Diverse desires** **Diverse beliefs** *Knowledge access* *Content false belief* *Hidden emotion*	TH = DHH TH = DHH HA TH > DHH CI TH > DHH	MHL > SHL	*

TH = typical hearing; DHH = deaf and hard-of-hearing individuals; HP = hearing parents; DP = deaf parents; DoDP = deaf individual of deaf parents; DoHP = deaf individual of hearing parents; Ex = experiment; NR = not reported; HD = hearing devices; EarlyHD = early experience with hearing devices; LateHD = late experience with hearing devices; HA = hearing aids; CI = cochlear implant; Early CI = early cochlear implantation; Late CI = late cochlear implantation; Dsibling = deaf sibling; Hsibling = hearing sibling; Dfamilily = deaf family; BI = bilateral hearing device; UNI = unilateral hearing device; SL = sign language; EarlySL = early access to sign language; LateSL = late access to sign language; D = deaf; bl school = school that use oral and SL; SL school = school that use only SL; oral school = school that use only oral language; DHHCI = deaf and hard-of-hearing individuals users of cochlear implants; DHHHA = deaf and hard-of-hearing individuals users of hearing aids; y = years. Visual coding scheme: bold = No differences in performance; black no bold = differences in performance; italics = mixed findings; grey = no information about performance; MHL = mild to moderate hearing loss; SHL = severe-to-profound hearing loss; * = low risk of bias; ** = moderate risk of bias; *** = high risk of bias. When the age interval was not available, the mean age was reported.
